# Continuous Remote Patient Monitoring in Patients With Heart Failure (Cascade Study): Protocol for a Mixed Methods Feasibility Study

**DOI:** 10.2196/36741

**Published:** 2022-08-25

**Authors:** Courtney Reamer, Wei Ning Chi, Robert Gordon, Nitasha Sarswat, Charu Gupta, Safwan Gaznabi, Emily White VanGompel, Izabella Szum, Melissa Morton-Jost, Jorma Vaughn, Karen Larimer, David Victorson, John Erwin, Lakshmi Halasyamani, Anthony Solomonides, Rema Padman, Nirav S Shah

**Affiliations:** 1 Department of Medicine NorthShore University HealthSystem Evanston, IL United States; 2 Outcomes Research Network NorthShore University HealthSystem Evanston, IL United States; 3 Department of Medicine Pritzker School of Medicine University of Chicago Chicago, IL United States; 4 Department of Family Medicine NorthShore University HealthSystem Evanston, IL United States; 5 Home and Hospice Services NorthShore University HealthSystem Evanston, IL United States; 6 physIQ, Inc Chicago, IL United States; 7 Department of Medical Social Sciences Northwestern University Evanston, IL United States; 8 Heinz College of Information Systems and Public Policy Carnegie Mellon University Pittsburgh, PA United States

**Keywords:** continuous remote patient monitoring, remote patient monitoring, feasibility, heart failure, wearable biosensor, preliminary efficacy, mobile phone

## Abstract

**Background:**

Heart failure (HF) is a prevalent chronic disease and is associated with increases in mortality and morbidity. HF is a leading cause of hospitalizations and readmissions in the United States. A potentially promising area for preventing HF readmissions is continuous remote patient monitoring (CRPM).

**Objective:**

The primary aim of this study is to determine the feasibility and preliminary efficacy of a CRPM solution in patients with HF at NorthShore University HealthSystem.

**Methods:**

This study is a feasibility study and uses a wearable biosensor to continuously remotely monitor patients with HF for 30 days after discharge. Eligible patients admitted with an HF exacerbation at NorthShore University HealthSystem are being recruited, and the wearable biosensor is placed before discharge. The biosensor collects physiological ambulatory data, which are analyzed for signs of patient deterioration. Participants are also completing a daily survey through a dedicated study smartphone. If prespecified criteria from the physiological data and survey results are met, a notification is triggered, and a predetermined electronic health record–based pathway of telephonic management is completed. In phase 1, which has already been completed, 5 patients were enrolled and monitored for 30 days after discharge. The results of phase 1 were analyzed, and modifications to the program were made to optimize it. After analysis of the phase 1 results, 15 patients are being enrolled for phase 2, which is a calibration and testing period to enable further adjustments to be made. After phase 2, we will enroll 45 patients for phase 3. The combined results of phases 1, 2, and 3 will be analyzed to determine the feasibility of a CRPM program in patients with HF. Semistructured interviews are being conducted with key stakeholders, including patients, and these results will be analyzed using the affective adaptation of the technology acceptance model.

**Results:**

During phase 1, of the 5 patients, 2 (40%) were readmitted during the study period. The study completion rate for phase 1 was 80% (4/5), and the study attrition rate was 20% (1/5). There were 57 protocol deviations out of 150 patient days in phase 1 of the study. The results of phase 1 were analyzed, and the study protocol was adjusted to optimize it for phases 2 and 3. Phase 2 and phase 3 results will be available by the end of 2022.

**Conclusions:**

A CRPM program may offer a low-risk solution to improve care of patients with HF after hospital discharge and may help to decrease readmission of patients with HF to the hospital. This protocol may also lay the groundwork for the use of CRPM solutions in other groups of patients considered to be at high risk.

**International Registered Report Identifier (IRRID):**

DERR1-10.2196/36741

## Introduction

### Background

Heart failure (HF) is a growing global public health concern. Worldwide, the estimated prevalence of HF has increased to >37.7 million cases, and in the United States the estimated prevalence is 6.5 million cases [1,2]. HF is associated with increased mortality, morbidity, and loss of quality-adjusted life years [3]. HF also has a significant economic burden; it is estimated that the overall cost of HF in the United States was US $30.7 billion in 2012, and the total cost of HF hospitalizations in the United States was US $11 billion in 2014 [4,5]. HF is a leading cause of hospitalizations among adults in the United States, and Medicare patients with HF have the highest readmission rates, ranging from 17% to 28.2% [3,5].

HF readmissions have become an increasing focus of quality improvement, and many readmissions are viewed as preventable [4,5]. The Affordable Care Act initiated the Hospital Readmission Reduction Program, which imposes a financial penalty on hospitals with excess 30-day unplanned readmissions [6]. Although these measures have been somewhat effective, additional strategies are necessary to continue improvement in this metric [5]. A promising strategy for improving outcomes in HF, including readmissions, is the use of mobile health (mHealth) solutions.

### Related Works

The use of mobile apps is an area of mHealth strategies that have the potential to improve care for patients with HF. Patient-facing apps that focus on self-care and self-monitoring constitute one such area of interest. A meta-synthesis of mobile apps in cardiovascular disease found that mobile apps can help to improve modifiable risk factors for cardiovascular disease [7]. Schmaderer et al [8] developed a mobile app that enabled patients to record their daily weight and medication and provided patients with reminders and educational tips. The authors completed a randomized 3-arm trial that found that patients randomized to the mobile app group or the mobile app plus internet-based–visit group had a trend toward improvement in health-related quality of life [8]. Another study performed a randomized controlled trial on the impact of a mobile app that promoted self-management and daily self-monitoring and found a statistically significant improvement in the Minnesota Living with Heart Failure Questionnaire score at 6 weeks but not at 12 weeks [9]. Overall, interventions using mobile apps alone, without other support built in, have had limited success in improving outcomes in patients with HF [8,9]. However, mobile apps have the potential to improve health, given their prevalence and portability, as well as their ability to record data, connect people, monitor activity, and provide patient-centric health care solutions. Ultimately, there is a further need for systemic assessment of potential mobile apps [10]. In addition, it is possible that mobile apps built in as part of an mHealth solution, as opposed to being the sole intervention, might lead to a more significant improvement in health-related outcomes.

Another possible mHealth approach for improving HF outcomes is remote patient monitoring. These solutions include the use of wearable or implanted devices, mobile apps, or other electronic devices that transmit data to health care providers. Implantable devices have shown potential as one such option for remote patient monitoring. The CardioMEMS Heart Sensor Allows Monitoring of Pressures to Improve Outcomes in NYHA Functional Class III Heart Failure Patients trial evaluated the use of CardioMEMS, an implanted pulmonary artery pressure–monitoring device, and demonstrated a reduction in morbidity, mortality, and hospitalization rate in patients with HF [11]. Hindricks et al [12] performed a randomized controlled trial evaluating the use of implant-based multiparameter telemonitoring compared with usual care in patients with new implantable cardioverter defibrillators or cardiac resynchronization therapy defibrillators and found that the telemonitoring group had a lower mortality score. Overall, some invasive remote monitoring solutions have demonstrated improvements in mortality [11,12] and a decrease in health care costs [13], but they have the large disadvantage of the need for an implantable device, leaving a gap for less-invasive monitoring.

Several studies have investigated forms of noninvasive remote monitoring and their efficacy in improving outcomes in HF [14-30]. A study showing a benefit was the Telemonitoring in the Management of Heart Failure study, a randomized controlled trial that evaluated a remote monitoring device that measured body weight, blood pressure, and heart rate [17]. These parameters were obtained daily, and email alerts were sent to providers when predefined criteria for interventions were met. This intervention, compared with usual care, reduced mortality, number of days lost to hospitalization, and death [17]. Overall, results of noninvasive remote monitoring studies have been mixed, with some showing a benefit in reducing mortality [15,17,30], improvement in quality of life [14,19,24,25,28], and reduction in readmissions [15,18,23,24,28,30], whereas some showed an improvement in outcomes [9,23,24]. Nonetheless, most remote patient monitoring solutions have yet to take advantage of recent advances in biosensor devices and machine learning technologies and thus do not provide intelligent continuous patient monitoring. Furthermore, raw data are typically collected from patients and required to be funneled through already overtaxed clinical providers [31]. In addition, many of the noninvasive remote monitoring solutions for patients with HF have depended on data collected at discrete time points, as opposed to continuously collected data.

Data collected at discrete time points might limit insight into the patient’s health status and may not reflect their condition during activities of daily living. There is some evidence that continuous remote patient monitoring (CRPM) might improve outcomes for patients [32-35]. For example, Downey et al [32] compared continuous vital sign monitoring with discrete vital sign monitoring in patients hospitalized after surgery and found that the continuous remote monitoring of vital signs group had a shorter length of stay and had fewer readmissions than the discrete vital signs monitoring group. Several studies have demonstrated that noninvasive CRPM can be used to help predict readmissions in patients with HF [36-38]. For example, Anand et al [36] completed a nonrandomized, prospective trial of 314 patients with HF where they used vital signs collected from an external chest sensor to develop and validate an algorithm to predict decompensation in patients with HF. They found that the algorithm had 63% sensitivity and 92% specificity in the validation cohort [36].

The latest advance in CRPM is pairing it with advanced machine learning analytics. By using a patient’s continuous physiological data stream and applying machine learning analytics, it is possible to detect a change in health status that is unique to that patient and not measured against population norms. Promising data supporting this were presented in phase 1 of this study. We found that elevated respiratory rate for individual patients may be associated with readmission [39]. Furthermore, the Multisensor Non-invasive Remote Monitoring for Prediction of Heart Failure Exacerbation study found that with a sensitivity of 87.5% and specificity of 86%, the analytics were able to predict worsening HF (rehospitalization), with a median time between the initial notification of a variance in vital signs and readmission of 6.5 (IQR 4.2-13.7) days [38]. This suggests there may be time to intervene before decompensation and readmission.

### Study Development 

Because of the complex medical needs of patients with HF, it is thought that interventions to improve quality of life and health-related outcomes will need to be multipronged and complex [27,40]. Given this, small-scale feasibility pilot studies are useful to determine the feasibility of intricate interventions and allow for refinement of the intervention [41]. We developed an interest in the potential of mHealth solutions to improve outcomes in patients with HF and specifically wanted to study a CRPM system with machine analytics because we feel that this is an untapped area that has the potential to improve outcomes [32-36,38,39]. We therefore developed a cascading-alert continuous remote monitoring system. Because of the complexity of this intervention, we have opted to develop a multiphase pilot feasibility study, with plans for a separate efficacy trial at a later date after the system has been fine-tuned and determined to be feasible.

### Objectives

We hypothesize that a continuous noninvasive remote monitoring solution with machine learning analytics used in a population with HF will lead to an earlier and more accurate prediction of decompensation and help to prevent readmissions. Therefore, the objective of the Cascade study is to evaluate the feasibility and preliminary efficacy of a CRPM program at NorthShore University HealthSystem (NSUHS).

## Methods

### Study Design and Implementation

This is a prospective, mixed methods, nonrandomized, open-label feasibility study. Phase 1 (n=5) was the soft launch and has already been completed [39]. Phase 2 (n=15) is a calibration and testing period to evaluate, adjust, and optimize the alerting criteria, monitoring protocol, and workflows. Phase 3 (n=45) is the pilot period of the optimized study protocol. The study outcomes include feasibility and preliminary efficacy, as well as operational, process, and patient-related outcomes. Feasibility will be determined by evaluating provider and patient acceptability and satisfaction and by evaluating the study attrition and study completion rate. Provider and patient acceptability and satisfaction will be assessed through qualitative measures using the affective adaptation of the technology acceptance model (A-TAM) [42]. Preliminary efficacy will be determined by comparing the study group readmission rate with a retrospective cohort readmission rate.

The study will use the pinpointIQ (physIQ) solution to continuously remotely monitor patients with HF for 30 days after discharge. We will use rules-based and machine learning algorithms to analyze patients’ physiological data collected from the VitalPatch biosensor (VitalConnect) to identify patients potentially at risk of decompensation. A structured cascading escalation and management care pathway will be used to intervene on patients determined to be at risk for decompensation.

### Participants

Participants will be recruited at NSUHS, which is a 9-hospital integrated health system in Chicago and surrounding suburbs in Illinois, United States. The intervention will be implemented at Evanston Hospital, a 354-bed hospital located in the Chicago suburbs, which has a cardiac care unit and an advanced HF cardiology consult service.

### Recruitment

Eligible participants are recruited from patients hospitalized at NSUHS with an HF exacerbation. A daily enterprise data warehouse query is executed to identify patients hospitalized with an HF exacerbation who meet the eligibility determined by specific inclusion and exclusion criteria. Patients considered for participation have an HF diagnosis, have New York Heart Association functional class II to class IV symptoms, have received at least one dose of an intravenous (IV) diuretic during their hospitalization, have a plan for discharging with partnering home health services, speak English, and are in the top 50% of the patients stratified using NSUHS’s 30-day readmission risk–prediction model called the clinical analytics prediction engine [43]. Patients are not considered if they meet any of the exclusion criteria, which include having a CardioMEMS device; having an allergy to hydrocolloid gel adhesive; being pregnant; being on dialysis; or having a documented visual, cognitive, or physical impairment that would interfere with the ability to comply with the study procedures.

### Device and Notification Mechanisms

**Figure 1:**
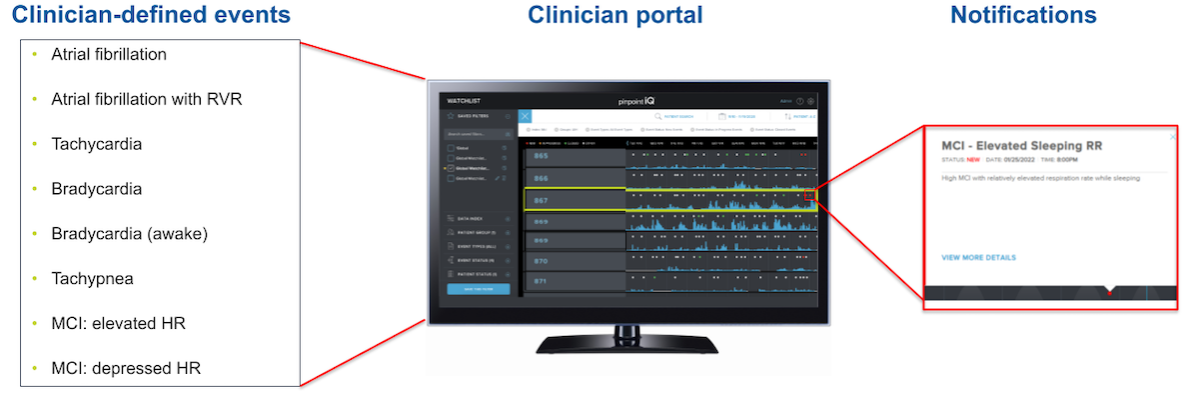
Overview of the clinician portal showing the portal, possible clinician-defined events, and a notification. HR: heart rate; MCI: multivariate change index; RR: respiratory rate; RVR: rapid ventricular response.

**Figure 2:**
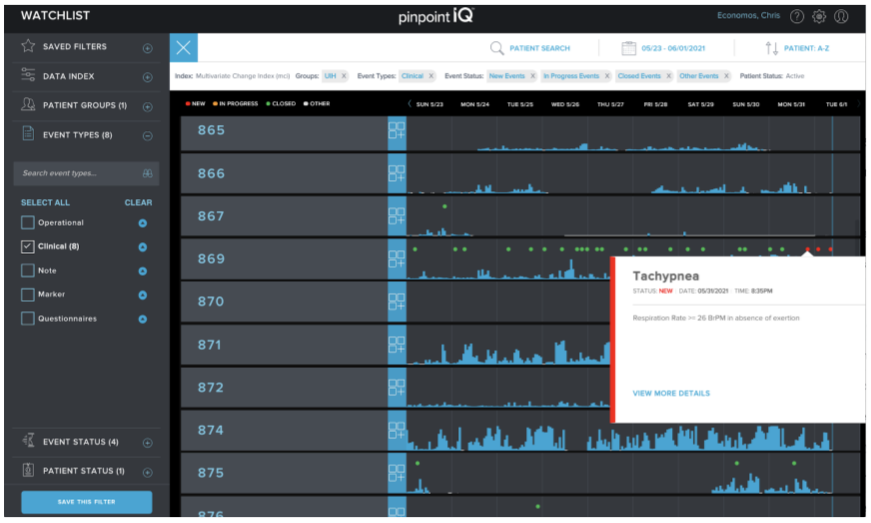
The clinician portal showing an alert and clinical events, where green dots represent events that have been seen already, and red dots represent new events.

The study uses the pinpointIQ solution, which includes Food and Drug Administration–cleared analytics that can provide early indication of patient deterioration and is capable of generating clinician-defined–event notifications. This is a closed loop monitoring solution that comprises a medical-grade biosensor with remote data collection capabilities, a smartphone app that acts as a data hub and electronic patient-reported outcome (ePRO) interface, a cloud computing platform for applying personalized analytics to patient data, a clinician portal for viewing biosensor data and analytics results that generate notifications (Figure 1), and an application programming interface. An overview of the clinician portal interface is shown in Figure 2.

Study participants are provided a chest-worn VitalPatch biosensor to wear, which collects near–real-time continuous ambulatory vital signs, including heart rate, respiratory rate, heart rate variability, activity level, sleep-wake determination, position, and atrial fibrillation detection (includes single-lead telemetry) once discharged. The biosensor is a disposable noninvasive patch that lasts 7 days and is replaced multiple times by the patient in the postdischarge period to have active monitoring for 30 days in total. This biosensor is a vendor medical device and has been tested separately from this study.

**Figure 3:**
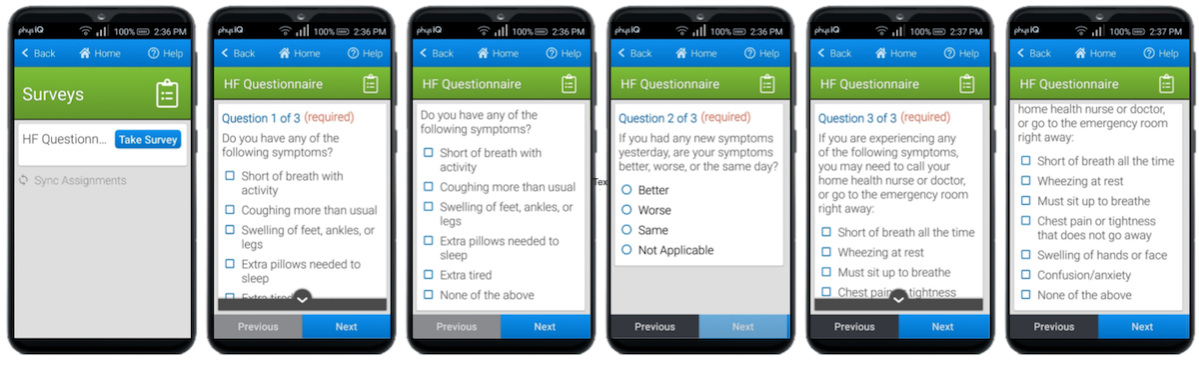
The daily symptom survey on the mobile app.

**Figure 4:**
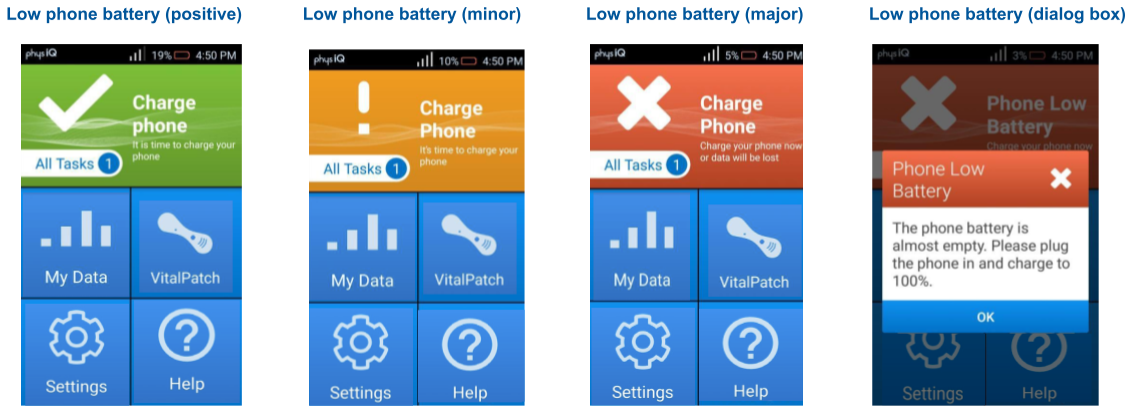
Mobile app alerts.

A study-specific smartphone is provided to the participant. The vendor developed an Android smartphone app that serves as the gateway for real-time data acquisition through Bluetooth from the biosensor. It also provides an interface for ePRO questionnaires (Figure 3). The vendor used data from focus groups of patients with chronic illness in the age range of the typical user to inform the smartphone app design. The app runs on a dedicated locked smartphone that is configured only to run the app, which has been validated to reliably interface with, and collect data from, the biosensor. The app interacts with the biosensor in real time and uploads data directly to the vendor platform over a secure cellular network connection. The app also provides patients with indications for proper data collection and escalating notifications of potential data loss, including connectivity issues, low battery, low memory, and unanswered questionnaires (Figure 4). If a patient is out of range with the smartphone, the data are still collected and stored by the VitalPatch biosensor for 8 hours. When the patient comes back into range with the smartphone, the collected and stored physiological data start uploading to the cloud again. The study smartphone’s battery life is approximately 12 hours, and the patients are trained to charge the study smartphone daily.

The patient is given a separate study smartphone as opposed to using their own mobile phone for a few reasons. First, many older patients with HF do not own a smartphone; providing them with a study smartphone helps to include patients with HF who may otherwise be excluded from the study. Second, the study smartphone only contains the vendor’s app; if the patient ever loses the study smartphone, the vendor can remotely wipe the smartphone’s data to ensure that patient privacy is protected. The study smartphone is slim and easy to carry, and most patients do not find having 2 mobile phones inconvenient.

**Figure 5:**
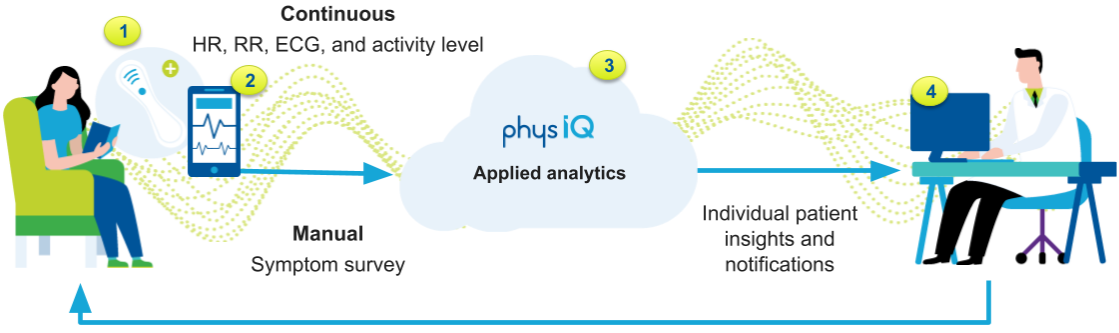
Data transmission pathway. ECG: electrocardiogram; HR: heart rate; RR: respiratory rate.

The physiological data are transmitted from the study smartphone to the cloud and analyzed by rules-based and machine learning algorithms to help identify risk of decompensation in patients with HF. The data transmission pathway is shown in Figure 5. Rules-based notifications include tachypnea, tachycardia, bradycardia, atrial fibrillation, and atrial fibrillation with rapid ventricular response. The multivariate change index (MCI), a machine learning–based notification, is also calculated from the physiological data [38]. The MCI is a nonspecific patient deterioration model that trains on the first 48 hours of a patient’s physiology and then measures the difference between expected and observed physiology and signals in the clinician portal when there is a significant difference in these measurements. The system specifically calculates an MCI in relation to heart rate and respiratory rate and evaluates for 3 different MCI notifications: MCI elevated heart rate, MCI depressed heart rate, and MCI elevated sleeping respiratory rate.

The patients also complete a daily symptom survey (Figure 3) on the study smartphone based on the criteria from the HF zone tool, which is transmitted to the clinician portal [44,45]. Physiological notifications and daily symptom survey data (ePROs) are visible to the home health nurse (HHN) on the clinician portal. If predetermined criteria from the physiological events and ePRO responses are met, a structured and predefined electronic health record (EHR)–based pathway of health care provider telephonic management is initiated. The HHN is the first human in the loop in the cascade. If HHN escalation criteria are met, advanced practice providers (APPs) and potentially HF specialists are notified based on the acuity, type of clinical event, and call roster.

### Workflow and Clinical Management

**Figure 6:**
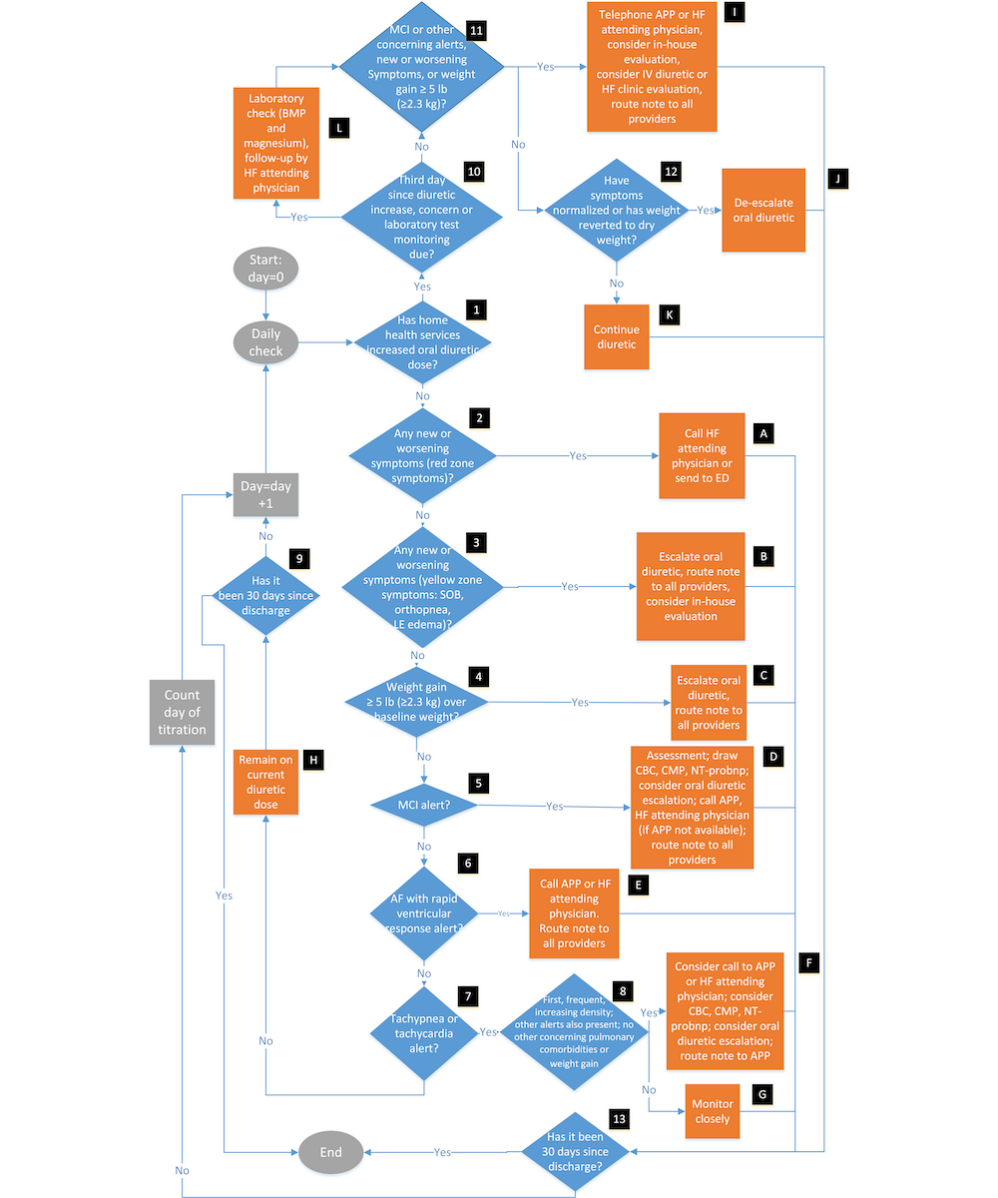
Workflow process map. AF: atrial fibrillation; APP: advanced practice provider; BMP: basic metabolic panel; CBC: complete blood count; CMP: comprehensive metabolic panel; ED: emergency department; HF: heart failure; IV: intravenous; LE: lower extremity; MCI: multivariate change index; NT-proBNP: N-terminal pro–B-type natriuretic peptide; SOB: shortness of breath.

The workflow process map is shown in Figure 6. The HHN reviews the web-based platform daily for any clinical event–driven notifications. The daily ePRO, which includes the patient’s daily weight and whether they are having symptoms related to HF, is also reviewed [44,45]. If a patient reports any symptoms on a particular day, the patient survey will also ask whether the symptoms are getting better, worse, or the same the following day.

If there are any red zone symptoms reported, which include symptoms of chest pain, shortness of breath at rest, wheezing at rest, swelling of the hands or face, confusion, anxiety, or feeling as though they must sit up to breathe, the HHN will call the HF specialist or send the patient to the emergency department (ED).

If there are any new or worsening symptoms of shortness of breath, orthopnea, or lower extremity edema (yellow zone symptoms), the HHN will call the patient and complete the structured EHR documentation note. The HHN documentation note provides automated management recommendations based on the information filled out in the note. In the case of a note stemming from new or worsening yellow zone symptoms, recommendations include increasing the oral diuretic dose and considering an in-house evaluation. The note will be forwarded to the APP pool and the patient’s HF specialist. The APP in turn will schedule a telephone visit with the patient and determine whether the patient needs home IV diuresis. If so, they will provide a prescription for this, and home health services will start IV diuretics at home. The HF attending physician and the HHN will be alerted of this plan.

If the patient has a weight gain of ≥5 lb (≥2.3 kg) compared with their baseline weight, the HHN will call the patient and complete the structured documentation note. Potential automated recommendations to consider include escalating the oral diuretic dose. The HHN will route the note to both the HF APPs and attending physician. The APP will follow up with the patient and schedule a telephone visit with the patient. The APP will assess whether the patient needs home IV diuresis, and if so, they will prescribe it. This plan will be communicated to the HF attending physician and the HHN.

If there is an MCI event, the HHN will call the patient and complete the structured note. Management recommendations include sending a visiting HHN to draw a complete blood count, a complete metabolic panel, and an N-terminal pro–B-type natriuretic peptide, as well as possible oral diuretic dose escalation. The HHN will also call the APP and route their note to the APP and HF attending physician. The APP will schedule a telephone visit with the patient and follow up on any laboratory test results. On the basis of their telephone visit, the patient’s ePROs, and any laboratory test results obtained, the APP will form a clinical assessment regarding the patient. On the basis of this assessment the APP will determine whether any of the following is indicated: an escalation of the oral diuretic dose, an urgent in-person clinic visit, and whether the HF attending physician needs to be alerted.

If there is an atrial fibrillation with rapid ventricular response event, the HHN will call the patient and complete the structured EHR note, call the APP or HF specialist, and route the note to the APP pool and HF specialist, or send the patient to the emergency room. If the APP is alerted to an atrial fibrillation with rapid ventricular response event, they will review the vendor platform to assess the event and the patient’s vital signs obtained from the biosensor. They will also schedule an urgent telephone or video visit with the patient to further assess them. The APP will determine whether the patient needs to be sent to the ED or whether the patient can be safely managed at home. The APP will also discuss their assessment with the HF attending physician to ensure that they agree with the plan.

If there is a tachypnea or tachycardia event, the HHN will evaluate whether this is the first time such an event has occurred or whether there is any increasing occurrence of these events. If so, the HHN will call and assess the patient and complete the structured note. Recommendations for management include calling the APP and HF specialist, considering conducting basic laboratory tests, and considering an oral diuretic escalation. If the APP or HF specialist is contacted, they will review the vendor platform and have a telephone visit with the patient. On the basis of their clinical assessment, they will consider an oral diuretic dose increase and determine whether the patient needs to be seen for an in-person visit. The telephone visit assessment will be sent to the HHN.

If the patient’s diuretic dose has been escalated or if they are on IV diuretics, the patient’s laboratory test results will be checked on the third day of taking an escalated dose. If a patient on an already escalated diuretic dose has an event notification, new or worsening symptoms, or increased weight gain, the HHN will call the APP or HF specialist for further guidance. Recommended interventions include a home evaluation, an urgent HF clinic evaluation, or an IV diuretic at home. A patient’s escalated diuretic dose will be de-escalated once their symptoms have resolved or their weight has reverted to their dry weight (normal weight without any extra fluid in the body).

If a patient has >1 alert, the pathway for the alert deemed more severe will be followed. The severity of alerts in order from more severe to less severe are as follows: any new or worsening red zone symptoms, any new or worsening yellow zone symptoms, weight gain of ≥5 lb (≥2.3 kg), an MCI alert, an atrial fibrillation with rapid ventricular response alert, and a tachypnea or tachycardia alert. For example, if the patient has a new red zone symptom and an MCI alert, the pathway for the red zone alert will be followed. The more severe alerts are depicted higher up on the workflow shown in Figure 6.

When the HHN escalates care to the APP or HF specialist, suggested management and treatment plans will be available to the clinical care team, but nuanced clinical judgment is ultimately left to the provider in managing these patients with complex medical needs.

### Data Collection and Management

Clinical data, such as demographics, comorbidities, laboratory test results, procedures, medications, communications, office visits, and hospitalizations, as well as outcomes, such as 30-day readmission rates, for each patient are collected from the EHR. CRPM data, including raw physiological and operational data, clinical and operational notification data, and ePRO data, are collected from the vendor platform. All study information is being stored on NSUHS’s password-protected encrypted computers and password-protected servers.

Patients are given a unique study record number. The unique study record number is different from their medical record number. This unique record number is used to identify the patient on the vendor web platform. In addition to the study data, the study team collects patient name, medical record number, date of birth, and telephone number for the purpose of conducting the study and links this information to the unique study record number. This information and the key that links the unique study record number to the patient is stored on NSUHS’s secure server and only shared with the HHNs and the HF team involved in the study for patient care management. No personal health information is provided to the vendor or other study team members outside of NSUHS. All patient information is aggregated during study analysis, and no identifiers will be provided in the analysis. Upon study completion all study data will be destroyed, and verification will be provided to data governance.

During informed consent and enrollment, the study coordinator makes sure that patients are alone or in a room with family members who the patient agrees can participate in decision-making around the study. The door is closed, and the collection of any study-related information is paused when other staff or visitors enter the room. We allocate 1 hour for consent, and if required, we can extend the amount of time to make sure that the patient has all their questions answered and privacy maintained.

### Key Outcomes Measures and Statistical Analysis

We will conduct a mixed methods evaluation of the feasibility of the CRPM solution after the completion of all 3 phases of the study. Our primary end point will be to determine the feasibility of a 30-day CRPM solution and a cascading notification system. Feasibility will be assessed by evaluating the study completion rate and the study attrition rate between consent and end of the intervention and by evaluating provider and patient acceptability and satisfaction using the A-TAM [42]. We will use interviews to evaluate both patients’ and providers’ degree of technology acceptance; perceptions of, and satisfaction with, wearable biosensor patches; and satisfaction regarding escalation pathways. We will then carry out a directed content analysis of interview transcripts to identify specific themes informed by the A-TAM to help guide future implementations of continuous remote monitoring systems [46]. Specifically, researchers will conduct semistructured interviews with providers and patients. Different researchers will then analyze the interview transcripts and, using the A-TAM framework as a guide, they will identify significant phrases that represent each construct from the A-TAM. We will then develop a synopsis of the significant themes and their relationship to A-TAM constructs.

Preliminary efficacy will be determined by comparing the study group readmission rate with a retrospective cohort readmission rate. The retrospective cohort group will be created from patients with HF who meet the same inclusion and exclusion criteria and received usual care over the previous year. The patients in the control group will be matched based on demographics, discharge home with home health services, and clinical analytics prediction engine risk scores [43]. We will also evaluate whether continuous monitoring can improve care processes through an escalating feedback protocol by comparing clinical and outcome data in the CRPM group with those in a retrospective cohort control group. Comparative analysis will be performed using an interrupted time series design with a propensity-matched control group. The primary outcome measures and methods of evaluation are summarized in Table 1.

**Table 1:** Primary aims and outcome measures.

Primary aim and measurement		Method of evaluation
**Feasibility**		
	Provider acceptability	Pre-post interview and questionnaire
	Provider satisfaction	Pre-post interview and questionnaire
	Patient acceptability	Pre-post interview and questionnaire
	Patient satisfaction	Pre-post interview and questionnaire
	Attrition rate	Study data
	Completion rate	Study data
	30-day readmission rates	EHR^a^ query
**Preliminary efficacy**		
	Mortality	EHR query
	Self-care	Pre-post European Heart Failure Self-Care Behavior Scale [47]
	Quality of life	Pre-post Minnesota Living with Heart Failure Questionnaire [48]
	Self-efficacy	Pre-post Self-Care Self-Efficacy Scale [49]
	Social support	ENRICHD^b^ Social Support Inventory [50]

^a^EHR: electronic health record.

^b^ENRICHD: Enhancing Recovery in Coronary Heart Disease.

Secondary outcomes, including technical outcomes and process and operational outcomes, will also be assessed (Table 2). Technical outcomes include the usability of the wearable device, usability of the patient smartphone app, usability of the provider portal, and ease of use of the structured clinical HHN note. Operational and process metrics include reasons for attrition, patient adherence to daily weight, patient adherence to daily symptom survey, percentage of notifications responded to in 24 hours, and the number of protocol deviations compared with the total number of patient days. In addition, effective communication of ePROs and physiological signals from the technical platform to the various clinical providers will be assessed by recording significant events and process lapses. CRPM data, clinical and operational notification data, and ePRO data will be analyzed and summarized using standard statistical tests of mean, median, SD, and IQR for continuous measures and count and percentage for categorical measures.

In addition to self-developed questionnaires targeting patient and provider experience in the study, we will also use several validated questionnaires to assess a patient’s baseline values and changes before and after the study regarding self-care, quality of life, and social support. Specifically, we will use the following questionnaires:

1. Self-Care Self-Efficacy Scale: a scale to assess a patient’s thoughts regarding self-care [47]
2. Minnesota Living with Heart Failure Questionnaire: a patient-oriented measure of the adverse effects of HF on a patient’s life [48]
3. European Heart Failure Self-Care Behavior Scale, 9-item version: a scale to measure HF self-care behaviors [49]
4. Enhancing Recovery in Coronary Heart Disease Social Support Inventory: a questionnaire to assess social support [50]

We will also identify valid patterns in the continuous remote monitoring patient data that may be associated with events of interest, such as escalation to IV diuretic at home, escalation to HF specialist, 30-day readmission rates, and ED presentation, using temporal pattern mining and feature extraction methods. Unstructured data in patient reports and clinician notes will be analyzed using simple text analysis methods, including text preprocessing (eg, tokenization and lemmatization) to infer sentiments and extract features in the texts that may be relevant for the events for interest (eg, term frequency–inverse document frequency and term frequency). As this is a feasibility study with a limited sample of patients, we would not have sufficient power to determine statistical significance of the results; therefore, we are not in a position to create a power calculation.

**Table 2:** Secondary aims and outcome as well as operational and process measures.

Secondary aim and measurement		Method of evaluation
**Technical outcomes**		
	Usability of the wearable device	Interviews and questionnaires
	Usability of the patient smartphone app	Interviews and questionnaires
	Usability of the provider portal	Interviews and questionnaires
	Ease of use of the structured clinical HHN^a^ note	Interviews and questionnaires
**Operational and process outcomes**		
	Reasons for attrition	Study data
	Patient adherence to daily weight	Study data
	Patient adherence to daily symptom survey	Study data
	Percentage of notifications responded to in 24 hours	Study data and EHR^b^ query
	Number of protocol deviations per number of patient days	Study data

^a^HHN: home health nurse.

^b^EHR: electronic health record.

### Ethics Approval

The NSUHS Institutional Review Board reviewed and approved this study (EH20-288).

## Results

### Phase 1

Phase 1 started in December 2020, and the last patient completed the study in March 2021 [39]. Phases 2 and 3 started in April 2021 and are estimated to be completed by the end of 2022.

During phase 1, we enrolled 5 patients, and the results are described in a separate paper [39]. The results of phase 1 are summarized herein. Of the 5 patients, 2 (40%) were readmitted during the course of the study; of these 2 patients, 1 (50%) was readmitted with an HF-related issue and 1 (50%) was readmitted because of an infection. Patient 101 was adherent to completing the daily survey and daily weights 70% of the time, patient 102 was adherent 83% of the time, patient 103 was adherent 90% of the time, patient 104 was adherent 93% of the time, and patient 105 was adherent 12.5% of the time. In total, there were 128 clinical alerts during phase 1. Of the 5 patients, 1 (20%) had atrial fibrillation and bradycardia alerts, 4 (80%) had tachypnea alerts, and 3 (60%) had MCI alerts. The HHN responded to 99.2% (127/128) of the clinical alerts. The observed activity for each patient day was compared with the expected activity to determine the amount of protocol deviations. In total, 57 protocol deviations out of 150 possible patient days were observed in phase 1 of the study.

The results of phase 1 were analyzed, and the study protocol was optimized [39]. During phase 1, the protocol only had tailored recommendations for management for the MCI alert, which led to difficulty in determining what action to take for other clinical alerts. Therefore, the protocol was updated to include tailored recommendations for additional alerts. It was noted that the MCI alert was generated only 1 day before admission for 20% (1/5) of the patients and during admission for another patient. The vendor therefore updated the MCI to make it more sensitive. In addition, the survey used during phase 1 was found to have an insufficient characterization of HF symptoms. Therefore, the study protocol was changed to instead use a validated existing HF symptom survey [44,45]. In addition, originally the study workflow was designed for the HHN to communicate with the HF registered nurse, but it was discovered that the HF registered nurses did not feel comfortable with deciding on patient management. Therefore, the study workflow was redesigned to have the HHNs communicate with the HF APPs and attending physicians. Furthermore, the HF APPs were sometimes unsure of what their response to clinical alerts should be; therefore, specific workflow and recommendations were built out for them.

### Phases 2 and 3

Phases 2 and 3 are ongoing; therefore, results are not yet available. Once completed, the data and results from phases 2 and 3 will be published in peer-reviewed scientific journals.

## Discussion

### Overview

The Cascade study is a feasibility study using an innovative CRPM solution with applied machine learning analytics, linked to an escalating cascading notification system with structured interventions in patients with HF. In this study, patients wear a chest-worn biosensor that collects continuous physiological data that are then analyzed by rules-based and machine learning algorithms to identify physiological perturbation. Patients also complete a daily symptom survey. If criteria based on the physiological data and survey answers are met, notifications are triggered, and a predetermined telephonic management workflow is pursued. As far as we know, this is the first study that integrates CRPM with both machine learning algorithms to provide providers with notifications of physiological decompensation and a cascading notification system and structured intervention protocols to manage patients after discharge.

The Cascade study is unique in that it evaluates the feasibility of a continuous remote monitoring system with applied machine learning analytics, as opposed to random spot checks of physiological data. Studies have shown that continuous remote monitoring compared with intermittent monitoring in patients admitted to hospital can lead to earlier detection of clinical deterioration and improve patient outcomes [32-35]. We believe that the use of a continuous monitoring system in the postdischarge period may lead to earlier detection of patients at risk for decompensation and may also reduce readmission rates in an ambulatory population with HF.

A unique attribute of this study is the cascading notification system to efficiently identify and communicate with the right provider required to make management decisions. The HHN is the critical gatekeeper in the cascading system. They collect information from the platform and patient and enter it into a structured EHR note that automates recommendations on whether management requires escalation to a different provider. If escalation is required, it targets either the APPs or the HF physicians or both providers. As CRPM has the potential for alert fatigue given the high volume of data collected and analyzed, the cascading system allows for each provider to function at their level of expertise and spreads the clinical decision-making to the right provider at the right time.

The automated EHR note is another innovation within this CRPM pathway. As noted earlier, the HHN assessment in response to notifications includes calling the patient and filling out a structured EHR note. The note was designed for the Cascade study; not only does it orchestrate the cascading system, but it also provides automated recommendations for interventions based on the data elements filled in by the HHN. Ultimately, it guides the HHN to ask the most appropriate questions to the patient and target the patient with a personalized set of interventions, thereby empowering a general HHN to take part in actively managing patients with HF with complex medical needs, while escalating to the most appropriate provider in the most efficient manner when necessary. As was noted in the soft launch, these structured intervention protocols were also enhanced through key learnings on how to interpret continuous data and notifications, which allows for standardization of the initial set of HHN management decisions. We have also created suggested management recommendation workflows for APPs and HF physicians but have left ultimate clinical management to the clinical providers, as described in the Workflow and Clinical Management section.

Another strength of this study is its 3-phase nature, allowing for a soft launch period in the study design to evaluate the CRPM solution on a small number of enrolled participants and a calibration and testing period before the pilot [39]. The soft launch allowed the research team to evaluate the study protocol, notifications, communication pathways, and workflows, as well as make rapid iterative changes [39]. The calibration and testing period is to fine-tune the physiological algorithms, notification scheme, and workflows before arriving at a final fixed state.

### Limitations

This study is limited because it is a feasibility study with a population of limited size. More studies with a larger sample size will have to be completed to determine the effectiveness of a remote monitoring solution in preventing hospital readmissions in patients with HF. The study is also limited in that only patients who are eligible and participate in partnering home health services programs are eligible for the study. This decreases the generalizability of this study to other patient populations.

### Future Works

This study will enable evaluation of a CRPM program in patients with HF and allow for improvement and modifications to the intervention. This study will pave the way for a larger efficacy trial to determine the effectiveness of a CRPM solution in patients with HF after discharge. In addition, this study will help to create a general framework for CRPM workflow and inform the development and implementation of CRPM programs for other populations categorized as high risk and receiving postacute care.

### Conclusions

The results from this study will determine the feasibility of a noninvasive remote monitoring system in recently discharged patients with HF. This study will use multiple validated and self-developed questionnaires and qualitative interviews to assess how this CRPM solution affects patients’ self-care, quality of life, and social support. This study’s findings will also help to aid earlier detection of patients with HF who are at risk for decompensation. In addition, this study will elicit both provider and patient feedback regarding the use of a remote monitoring system, which will help to determine key stakeholder perceptions regarding the use of CRPM systems and escalation and workflow pathways.

## Abbreviations

APP: advanced practice provider
A-TAM: affective adaptation of the technology acceptance model
CRPM: continuous remote patient monitoring
ED: emergency department
EHR: electronic health record
ePRO: electronic patient-reported outcome
HF: heart failure
HHN: home health nurse
IV: intravenous
MCI: multivariate change index
mHealth: mobile health
NSUHS: NorthShore University HealthSystem
